# Effect of hypoxia on the retina and superior colliculus of neonatal pigs

**DOI:** 10.1371/journal.pone.0175301

**Published:** 2017-04-13

**Authors:** Noelia Ruzafa, Carmen Rey-Santano, Victoria Mielgo, Xandra Pereiro, Elena Vecino

**Affiliations:** 1 Department of Cell Biology and Histology, University of Basque Country UPV/EHU, Leioa, Vizcaya, Spain; 2 Research Unit for Experimental Neonatal Respiratory Physiology, Cruces University Hospital, Barakaldo, Vizcaya, Spain; Universidade Federal do ABC, BRAZIL

## Abstract

**Purpose:**

To evaluate the effect of hypoxia on the neonatal pig retina and brain, we analysed the retinal ganglion cells (RGCs) and neurons in the superior colliculus, as well as the response of astrocytes in both these central nervous system (CNS) structures.

**Methods:**

Newborn pigs were exposed to 120 minutes of hypoxia, induced by decreasing the inspiratory oxygen fraction (FiO_2_: 10–15%), followed by a reoxygenation period of 240 minutes (FiO_2_: 21–35%). RGCs were quantified using Brn3a, a specific nuclear marker for these cells, and apoptosis was assessed through the appearance of active caspase-3. A morphometric analysis of the cytoskeleton of astrocytes (identified with GFAP) was performed in both the retina and superior colliculus.

**Results:**

Hypoxia produced no significant change in the RGCs, although, it did induce a 37.63% increase in the number of active caspase-3 positive cells in the superior colliculus. This increase was particularly evident in the superficial layers of the superior colliculus, where 56.93% of the cells were positive for active caspase-3. In addition, hypoxia induced changes in the morphology of the astrocytes in the superior colliculus but not in the retina.

**Conclusions:**

Hypoxia in the neonatal pig does not affect the retina but it does affect more central structures in the brain, increasing the number of apoptotic cells in the superior colliculus and inducing changes in astrocyte morphology. This distinct sensibility to hypoxia may pave the way to design specific approaches to combat the effects of hypoxia in specific areas of the CNS.

## Introduction

Neonatal hypoxic-ischemic brain injury is a prominent cause of neurological disability in neonates [1, 2]. Since it is not possible to conduct controlled studies in children, it is necessary to perform experimental studies in suitable animal species to obtain information that is likely to be applicable to humans. In this respect, pigs have for long been used as an experimental model given that many of their anatomical and physiological characteristics closely resemble those of humans, more so than other non-primate species [3–5].

The retina pertains to the central nervous system (CNS) and it is one of the most metabolically active tissues in the body [6]. Its high-energy demand is due to the highly sensitive and efficient system that converts light energy into neuronal signals, the reason why the retina consumes oxygen more rapidly than other tissues [7, 8]. Thus, in times of increased energy demand, oxygen becomes one of the most limited metabolites in the retina [9]. For this reason, the retina is susceptible to alterations in oxygen tension and specifically, the retina is sensitive to hypoxia, a condition defined as an inadequate supply of oxygen for an organism, tissue or cell [10]. At the cellular level, functional studies suggest that retinal ganglion cells (RGCs), the neurons that relay visual signals to the brain, may be the most sensitive cells in the retina to experimental transient ischemia or systemic hypoxia [11]. Indeed, a reduction in oxygen tension could be associated with the development of retinal pathologies, such as retinal vessel occlusion, proliferative diabetic retinopathy, retinopathy of prematurity, glaucoma, age-related macular degeneration or high altitude retinopathy [12]. Death of RGCs is a hallmark of retinal diseases in which hypoxia and/or ischemia are assumed to play an etiological role [13–16].

While the brain represents 2% of our body weight, it consumes 20% of the body’s oxygen demand. Moreover, the immature foetal and neonatal brains are particularly vulnerable to severe alterations in oxygen tension and they may develop neurovascular malformations when oxygen levels are low [17, 18]. However, in mammalian neonates certain physiological responses and adaptations exist to respond to a limited oxygen supply [19]. The superior colliculus is a multilayered structure in the mammalian midbrain, and it is the structure in the brain where among inputs from retinal axons and the visual cortex converge [20–23]. As hypoxia triggers apoptosis [24], it is often best to study this phenomenon by counting the number of recently activated apoptotic cells. Moreover, RGCs are sensitive to hypoxic conditions and the death of these cells provokes a gradual loss of vision that will ultimately lead to blindness. As such, we evaluated the apoptosis induced by hypoxia by examining the distribution of cells with active caspase-3 in the superior colliculus and retina, the activation of which is a marker of this form of cell death.

Glial cells play crucial roles in regulating neuronal development and neuronal activity, and astrocytes in particular are susceptible to reductions in oxygen tension [25]. Given their importance in linking vascular and neuronal function, astrocytes are fundamental in the induction of neuronal deficits, and hypoxia is known to induce astrocyte-dependent protection of neurons [26, 27]. They are the first cells exposed to the damage from hypoxic or ischemic insults [28, 29]. Moreover, hypoxia also affects the ability of astrocytes to sustain neuronal viability and it induces specific molecular responses in astrocytes [30]. Indeed, the degeneration of astrocytes is associated with the functional failure of the blood retinal barrier in oxygen-related neuropathies [29, 31]. For these reasons, we assessed the morphological changes to the cytoskeleton of astrocytes in the retina and superior colliculus. It is important to note that within the retina, another macroglial cell type is present in addition to astrocytes that are not present in the brain, the Müller glia. These cells are specialized in maintaining the homeostatic and metabolic support of retinal neurons, as well as fulfilling other functions [32]. In addition, there is some metabolic heterogeneity and distinct vulnerability to hypoxia within different tissues [33] that could be responsible for producing a distinct response to hypoxia of the tissues of interest here.

Given that hypoxia has a negative effect on neuronal metabolism and that it may be detrimental to the function of neurons, and since astrocytes have the capacity to sustain normal neuronal activity and to regulate the development of the vasculature, here we evaluated whether low oxygen conditions induce changes in neurons and astrocytes in the retina and superior colliculus.

## Materials and methods

### Animal preparation

This study was carried out in strict accordance with the recommendations for the Experimental Research Committee of the Cruces University Hospital, which is registered in the Official Register of Breeders, Suppliers and Users of animals for experimental and other scientific purposes in the Basque Country (Spain). All the experimental protocols met with the European (2010/63/UE) and Spanish (RD53/2013) guidelines for the protection of experimental animals and they were approved by the Ethics Committee for Animal Welfare of the Cruces University Hospital. On the same day as the experiment, animals were obtained from a local farm authorized by the Basque Country Regulatory Agency to supply animals for research. All animals were free of any disease and they were transported with a certificate of health.

The protocol to produce hypoxia used in the present study has been described extensively elsewhere [34]. Neonatal pigs (*Sus scrofa*, n = 8) aged 2–4 days old (1.7± 0.2 kg) were sedated with an intramuscular injection of ketamine (15 mg/kg) and diazepam (2 mg/kg). Before performing the surgical procedure, anaesthesia and analgesia were induced by intravenously administering fentanyl (5 μg/kg) and propofol (1.2 mg/kg), and this state was maintained by continuous intravenous infusion of fentanyl (titrated as necessary: 5–20 μg/kg/h), propofol (titrated as necessary: 2–3 mg/kg/h) and midazolam (titrated as necessary: 0.5–2 mg/Kg/h). In addition, the animals used as controls were paralysed by continuous intravenous infusion of vecuronium bromide (3 mg/kg/h). In all cases, an ear vein was catheterized to deliver the anaesthesia and analgesia. A tracheotomy was performed, and a tracheal tube (4.0 mm ID) was inserted and connected to a ventilator. Animals were then positive pressure ventilated and changing ventilator parameters were performed to maintain adequate blood gas values of arterial oxygen pressure (PaO_2_) 80–110 mmHg, adequate arterial pressure of carbon dioxide (PaCO_2_) 35–45 mmHg and a pH 7.30–7.45.

An arterial catheter was inserted into the femoral artery to monitor the mean arterial blood pressure (MABP) and heart rate (HR), and to obtain arterial blood samples for blood gas analysis: PaO_2_, PaCO_2_, pH, Base Excess (EB), oxygen saturation and lactic acid (GEM Premier 4000, Instrumentation Laboratory). Animals were also monitored continuously by three-lead electrocardiogram during the experimental procedure.

### Hypoxia

The hypoxia model is based on a swine model of neonatal asphyxia [35]. Two experimental groups were established, control (n = 4) and hypoxic (n = 4) pigs. In the hypoxic group, the animals were stabilized for 30 minutes and hypoxia was then induced by decreasing oxygen levels to 12–14% for 120 minutes, followed by 240 minutes of normoxia. Hypoxia was induced by reducing the fraction of inspired oxygen (FiO_2_, the fraction or percentage of oxygen in the space being measured) to 0.1–0.15 while increasing the concentration of inhaled nitrogen gas. Control pigs were not subjected to reduced oxygen concentrations. Following hypoxia, the FiO_2_ levels were increased to 21–35% in order to maintain normoxia for 240 minutes. At the end of the experiments (4 hours after the onset of hypoxic injury), the animals were sacrificed with an intravenous overdose of potassium chloride (0.35 mg/kg).

### Tissue collection

Neonates eyes were enucleated, and the cornea, crystalline lens and vitreous humour were removed. Each eyecup containing the retina was fixed overnight by immersion in 4% paraformaldehyde (PFA) in 0.1 M phosphate buffer (PB, pH 7.4) and after then washing in phosphate buffered saline (PBS, pH 7.4), the retina was carefully separated from the rest of the eye. In addition, the superior colliculus was extracted from the brain and immediately fixed overnight in 4% PFA. Control and hypoxic tissues were cryoprotected for 24 hours at 4°C in 30% sucrose diluted in 0.1 M PB, and the tissue was then embedded in OCT medium to obtain cryosections (14 μm thick) that were stored at −20°C.

### Immunohistochemistry

Whole mount retinas were immunostained as described previously [36], with some minor modifications. The retinas were washed with PBS and non-specific antibody binding was blocked by incubating them overnight at 4°C in a solution of PBS-TX-100-BSA (0.25% Triton-X 100 and 1% bovine serum albumin in PBS), with shaking. The retinas were then incubated (for one day with shaking at 4°C) with the primary antibodies diluted 1:1,000 in PBS-TX-100-BSA: an anti-Brn-3a goat polyclonal antiserum (Santa Cruz Biotechnology, Santa Cruz, USA) to detect RGC nuclei; and an anti-GFAP mouse monoclonal antibody (Sigma, Steinheim, Germany) to detect the cytoskeleton of astrocytes. Subsequently, the retinas were washed thrice in PBS for 15 minutes and antibody binding was detected (5 hours at room temperature with shaking) with secondary antibodies diluted 1:1,000 in PBS-BSA (1%): an Alexa Fluor 568 conjugated donkey anti-goat antibody (Invitrogen, Eugene, Oregon, USA) and an Alexa Fluor 488 conjugated rabbit anti-mouse antibody (Invitrogen, Eugene, Oregon, USA). Finally, the retinas were washed 3 times for 10 minutes in PBS, flat mounted onto slides in PBS-Glycerol (1:1) and coverslipped.

Cryostat sections of the retina and superior colliculus were immunostained as described previously [37]. After washing the sections twice in PBS-TX-100 for 10 minutes, they were incubated overnight with the primary rabbit anti-active (cleavage) caspase-3 (Asp175) antibody (1:1,600, Cell Signaling Technology #9661, Danvers, Massachusetts, USA) and an anti-GFAP mouse monoclonal antibody (1:1,000, Sigma, Steinheim, Germany). After washing twice in PBS, antibody binding was detected for 1 hour with an Alexa Fluor 555 conjugated goat anti-rabbit antibody (1:1,000, Invitrogen, Eugene, Oregon, USA) and an Alexa Fluor 488 conjugated goat anti-mouse antibody (1:1,000, Invitrogen, Eugene, Oregon, USA) diluted in PBS-BSA (1%). The sections were washed twice with PBS for 10 minutes and mounted with a coverslip in PBS-Glycerol (1:1).

### RGC quantification

To analyse the RGCs and astrocytes in the retina, confocal microscopy images of whole mount retinas were obtained at a resolution of 2048 x 2048 pixel with a 20X objective (Olympus FV500, Olympus, Tokyo, Japan). A Z-stack was obtained of five images with a 4 μm separation and a total of 8 whole mount retinas were studied (4 control and 4 hypoxic). Four different areas of the retina were analysed, the peripheral and central areas of the dorsal and ventral retina (S1 Fig). Thus, a total of 16 images from each retina were captured (four pictures of each analysed area). Since the porcine optic nerve is located ventrally, the dorsal area is larger than the ventral area, we selected the peripheral area 15 mm from the optic nerve in the dorsal domain and 6 mm from the optic nerve in the ventral domain. The central area refers to tissue 2 mm from the optic nerve in both the dorsal and ventral directions. These criteria were applied to the 8 retinas studied and thus, the total retinal area analysed was 8 mm^2^ per retina (2 mm^2^ per area). The method to quantify RGCs was slightly modified from that used previously [38], employing AxioVision 4.7.2.0.Software. The number of Brn3a labelled RGC nuclei in each retinal area of the experimental and control eyes was counted, and compared in the dorsal and ventral peripheral and central retina.

### Quantification of active caspase-3 positive cells

The cells with active caspase-3 were quantified in the retina and superior colliculus using a fluorescent microscope (Zeiss, Jena, Germany) and the Zeiss Zen software, analysing 5 sections from the same central and peripheral areas of each retina, and 20 sections from the same rostro-caudal level of each superior colliculus from 4 control and 4 hypoxic animals. Five images from each section were acquired at 20X on a Zeiss Axiocam MRM (Zeiss, Jena, Germany) and the active caspase-3 cells were counted double blind by two experimented researchers, as described previously [39]. In the retina, the active caspase-3 positive cells in the ganglion cell layer (GCL) were counted and the linear distance of each micrograph was measured (Zen, Zeiss, Jena, Germany). Thus, the number of labelled cells were expressed per linear millimetre of the RGC layer (cells/mm). In the superior colliculus, the number of active caspase-3 cells was counted in the superficial layers and in the total surface of the superior colliculus, including the superficial layers, calculating the average number of active caspase-3 positive cells per mm^2^ of the superior colliculus (cells/mm^2^).

### Astrocyte morphometry

To analyse the cytoskeletal morphology of astrocytes in the retina, images were taken of whole mount retinas using the same microscope and criteria as those used for RGC quantification. The astrocytes in the pig retina are organized mainly like those in the human retina, running parallel to the RGC axons. A semi-automatic method to measure astrocyte organization was used to measure the morphological changes in astrocytes. The aim was to quantify the differences in the astrocyte network between the control and hypoxic retinas based on the local orientation of the astrocytic processes. An *ad hoc* programme was developed in Matlab R2010b (MathWorks, Inc) to quantify the morphological changes to astrocytes. This programme estimates the local orientation of the GFAP-positive lines in an image, taking advantage of the SURF extraction algorithm [40] and its capacity to define local pixel orientation based on their neighbouring relationships. Following local characterization, a histogram of this orientation is extracted that allows the frequency of each direction to be measured (1-degree bins). This involves measuring the randomness of the histogram using Shannon’s Entropy equation [41], a well-known function to measure the predictability of a variable. The higher the entropy the less predictable and consequently, the more disorganized the system. With this information, the degree of disorganization of control astrocytes and hypoxic astrocytes can be compared. The average entropy from the histogram of the images was calculated and a statistical analysis was performed to evaluate our hypothesis that the entropy is greater in hypoxic retinal astrocytes.

To analyse the astrocytes in the superior colliculus, a Zeiss Axiocam MRM fluorescent microscope (Zeiss, Jena, Germany) and the Zeiss Zen software was used, obtaining 20 images (20X) at the same rostro-caudal level of the superior colliculus of the 4 control and 4 hypoxic animals. Since the astrocyte organization in the superior colliculus does not follow a well-established parallel pattern, the method used to analyse the astrocytes in this structure differed to that used in the retina. The morphology of the astrocyte cytoskeleton was analyzed using ImageJ (version 1.49), the image processing program developed at the National Institutes of Health (NIH). Using the “threshold color” tool, the region formed by colored pixels (labeled with the antibody against GFAP) was selected, and this area of the cytoskeleton of selected astrocytes was measured. Given the entire image area, we could calculate the proportion of the area occupied by astrocyte´s cytoskeleton in the control and hypoxic superior colliculus.

### Statistical analysis

RGC density, the number of active caspase-3 positive cells and the morphological data from the astrocyte’s cytoskeleton were described as the mean and standard error of mean, and these parameters were compared between control and hypoxic tissues. Statistical analyses were carried out using IBM SPSS Statistics software v. 21.0 and the homogeneity of the variances was assayed with Levene´s test (p < 0.05). To assess whether there were significant differences in the number of RGCs, the number of active caspase-3 positive cells and in astrocyte morphology between control and hypoxia conditions, a Student T-test was used. In addition, a Mann-Witney U test was used to verify the differences between the control and hypoxic groups. For both tests, the minimum value of significance was defined as p<0.05.

## Results

### Gas exchange

At baseline, all animals displayed adequate gas exchange, whereby the fraction of inspired oxygen (FiO_2_) was 30 ±2%, the PaO_2_ was 108 ±16 mmHg, PaCO_2_ was 42 ±6 mmHg, pH was 7.34 ±0.08, EB (base excess) was -2 ±3 mmol/l and lactic acid was 2 ±1 mmol/l. In the hypoxic animals, the decrease in oxygen levels produced a FiO_2_ of 12 ±2%, and a significant impairment of gas exchange, with a PaO_2_ of 31 ±8 mmHg, PaCO_2_ of 45 ±8 mmHg, pH of 6.84 ±0.05, EB of -24 ±2mmol/l and lactic acid of 17 ±2mmol/l. Moreover, the MABP (mean arterial blood pressure) decreased significantly after hypoxia (32 ±5 vs. 78 ±12 mmHg), while the HR (heart rate) remained unchanged relative to the basal values (184 ±36 vs. 196 ±33 bpm). These parameters partially reverted during the re-oxygenation period to a FiO_2_ of 33 ±1%, a PaO_2_ of 92 ±17 mmHg, a PaCO_2_ of 45 ±5 mmHg, a pH of 7.22 ±0.12, an EB of -8 ±6 mmol/l and lactic acid of 4 ±4 mmol/l. However, there was no change in either the MABP (36 ±9 mmHg) or HR (193 ±26 bpm). In the control animals that were not subjected to hypoxia, these parameters remained at the basal values throughout the experiment. Finally, the hemoglobin levels (7.0 ±0.7 g/dl) remained constant throughout the experiment and all pigs remained alive throughout.

### RGC density

**Fig 1:**
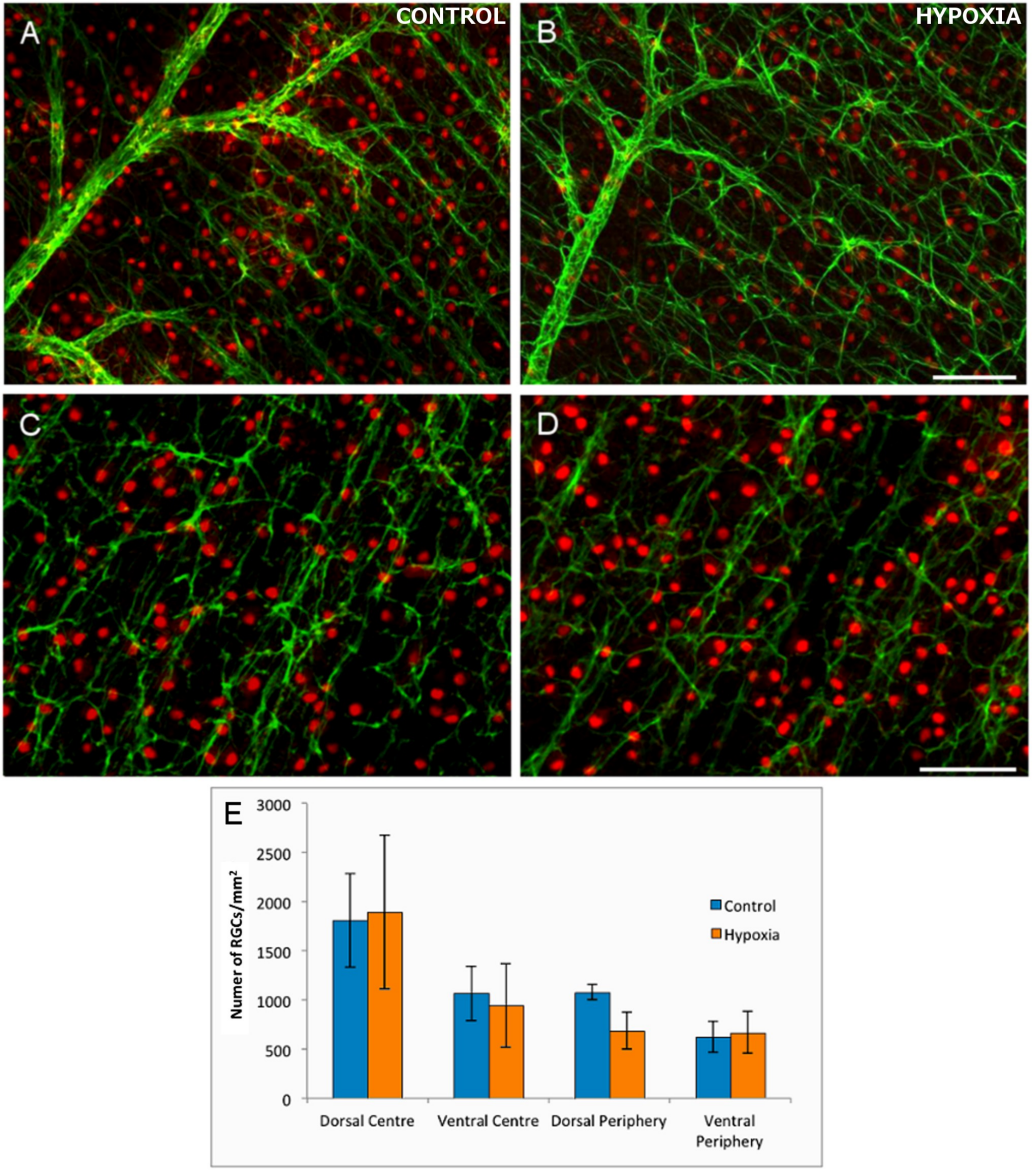
The analysis of flat mount retinas and RGCs. The cytoskeleton of astrocytes (green) are labelled with antibodies against GFAP and the nuclei of the RGCs (red dots) with antibodies against Brn3a in control (A, C) and hypoxic (B, D) retinas. Note the mild disorganization of the cytoskeleton in astrocytes and the reduction in the number of RGCs in hypoxic animals, although they are not significantly different from the controls. (E) Histogram representing the density of RGCs/mm^2^ in the four areas of the control and hypoxic retina analysed. Standard errors of the mean are represented as error bars. Scale bar = 100 μm.

The number of RGCs in the control and hypoxic retinas was analysed in the four selected areas (dorsal periphery, dorsal centre, ventral periphery, ventral centre: Fig 1A–1D). There were no significant differences in RGC number in the retinas from control and hypoxic animals in any of the four areas. While there was a mild tendency towards a loss of RGCs in the dorsal periphery of the hypoxic retinas, although this did not reach significance (Fig 1E and Table 1).

**Table 1:** Average RGC density. RGCs/mm^2^ ± standard error of the mean in the distinct areas of Control and Hypoxic retinas.

Domain	Control retina	Hypoxic retina
Dorso-peripheral	1079 ±75	686±19
Dorso-central	1808±48	1893±78
Ventral-peripheral	625±16	670±21
Ventral-central	1064±27	943±42

### Caspase-3 activation

**Fig 2:**
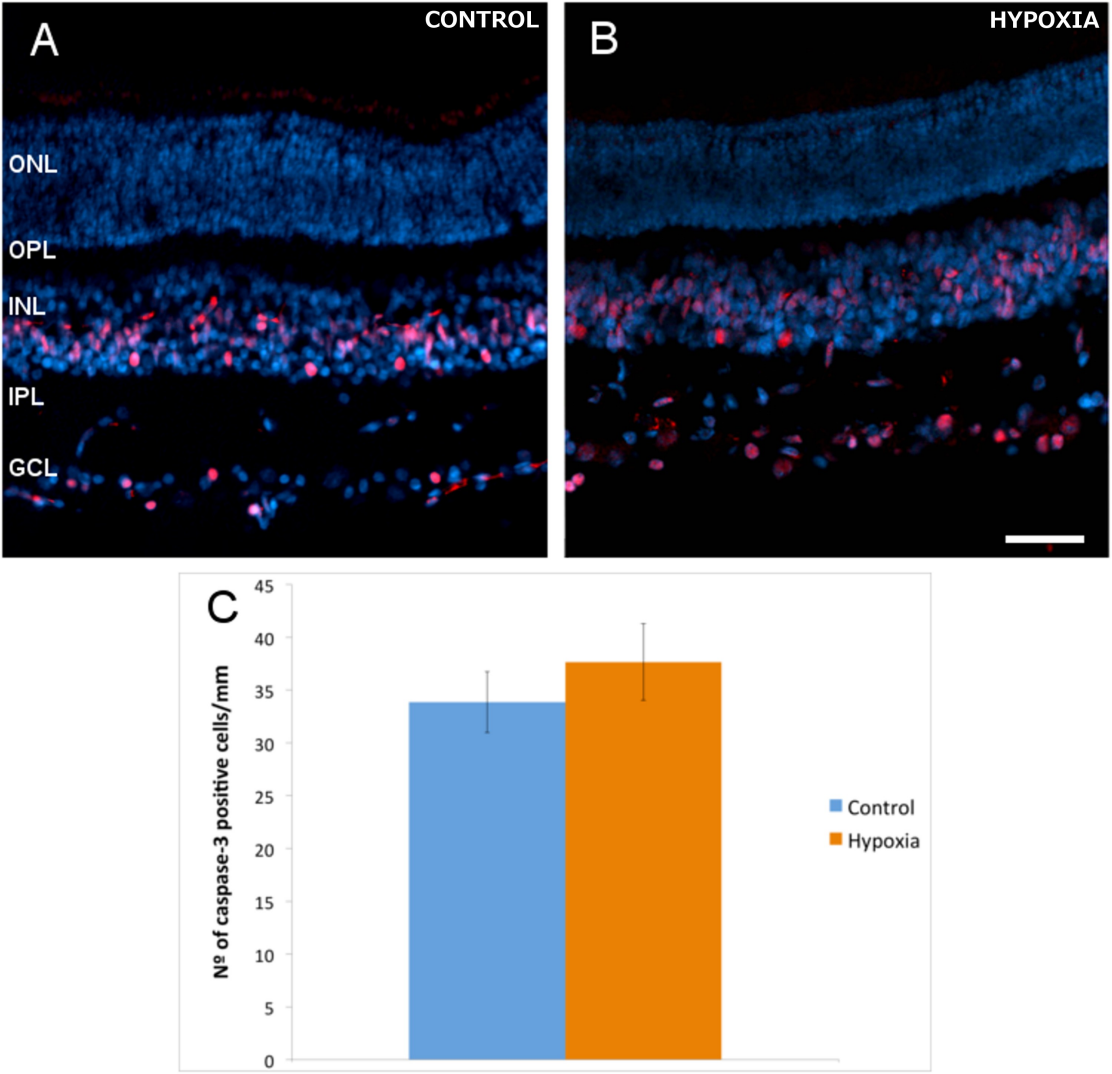
Active caspase-3-positive cells in retinal sections. The nuclei of neurons were labelled with DAPI (blue) and the apoptotic cells with an antibody against active caspase-3 (red) in control (A) and hypoxic (B) retinas. (C) Histogram of the average number of active caspase-3 positive cells per mm of the RGC layer in control and hypoxic retinas. The standard error of the mean is represented as an error bar: ONL, outer nuclear layer; OPL, outer plexiform layer; INL, inner nuclear layer; IPL, inner nuclear layer; GCL, ganglion cell layer. Scale bar = 50 μm.

**Fig 3:**
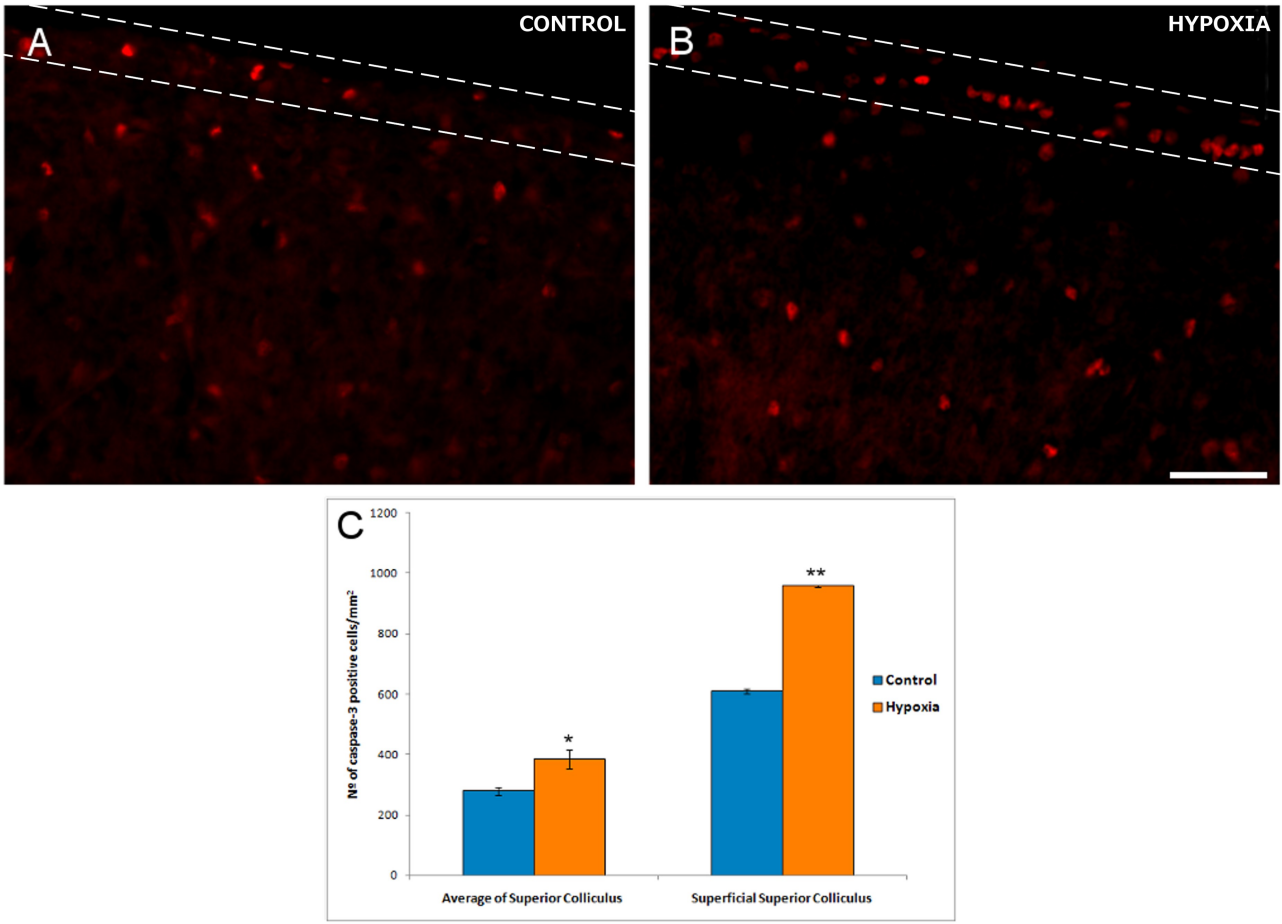
Active caspase-3-positive cells in sections of the superior colliculus. Images of the superior colliculus from control (A) and hypoxic (B) animals. Note that the superficial layers are defined by the dashed lines. Apoptotic cells are labelled with antibodies against active caspase-3 (red). Note the large number of active caspase-3 positive cells in the superficial layers of the superior colliculus. (C) Significant differences in the density of active caspase-3 positive cells between control and hypoxic pigs are shown in the histograms and the standard error of the mean is represented as an error bar: *p< 0.05, **p<0.01. Scale bar = 50 μm.

Numerous active caspase-3 positive cells were found in the inner nuclear layer (INL) and ganglion cell layer (GCL) of the retina, both in control and hypoxic animals. Indeed, hypoxia did not produce a significant change in the number of positive active caspase-3 cells in the GCL, with 34 (±3) active caspase-3 positive cells/mm in control retinas and 38 (±4) cells/mm in hypoxic retinas (Fig 2). By contrast there was a 37.6% increase in the number of active caspase-3 positive cells after hypoxia in the superior colliculus, rising from 279 (±13) cells/mm^2^ in the control animals to 384 (±30) cells/mm^2^ after hypoxia (p = 0.013, Fig 3). This increase was even stronger if only the superficial layers of the superior colliculus were taken into account, where the number of apoptotic cells increased by 56.93% with respect to the controls: 610 (±7) active caspase-3 positive cells/mm^2^ in the controls as opposed to 957 (±2) cells/mm^2^ in the hypoxic animals, (p = 0.002).

### Astrocyte morphology in the retina

**Fig 4:**
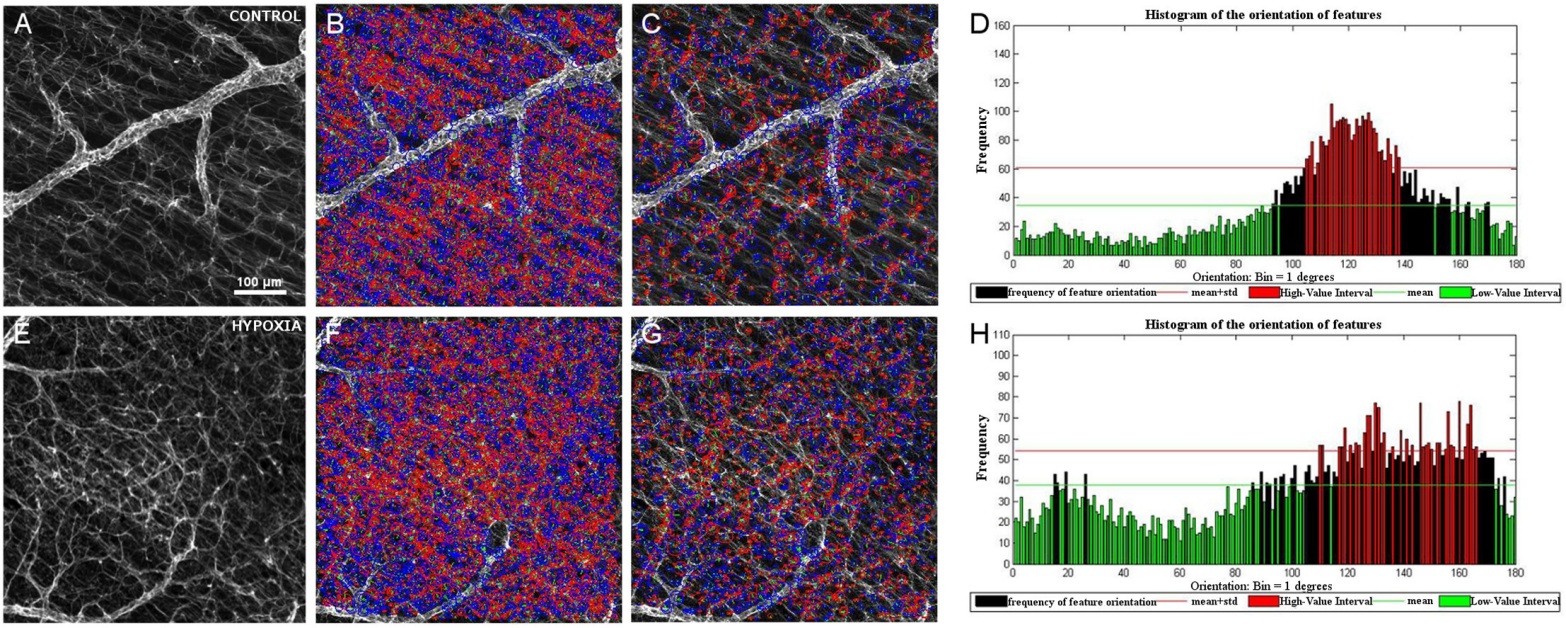
Astrocytes in the retina. Surf detection in GFAP labelled images of the control (A-D) and hypoxic (E-H) retina. GFAP immunohistochemistry (green) of the retina from control (A) and hypoxic animals (E) showing the relationship in the orientation of the astrocytes relative to the RGC axons (B, F). The relationship between the orientation of processes after removing the features corresponding to the main orientation (C, G). Histograms (D, H) quantifying the disorganization of the astrocytic processes using the Surf feature orientation for the input image: the higher the bar, the greater the number of features orientated in the direction indicated in the abscissa. The red bars indicate the highest number of events that take place at the angle indicated in abscises. Note that in the hypoxic histogram there are more red bars at different angles than in the control retinas. After analysing all the images, no significant differences between control and hypoxic retinas were found.

When we analysed the cytoskeletal morphology of astrocytes in the control and hypoxic neonatal pig retina, retinal astrocytes appeared to be more disorganised following hypoxia, with an increase in the lateral extension of processes (Fig 1). Using the algorithm described above, we quantified the disorganisation of the astrocyte networks in the retina, which was translated into different histograms of the orientations depending on the characteristics of the input (Fig 4). As such, some hypoxic retinas displayed a higher degree of randomness in the histograms, whereas control retinas seemed to have a greater difference in the frequency between the main orientation angles and other angles (Fig 4: parallel axon processes in red and non-parallel ones in green). The *ad hoc* routine to quantify the differences in the retinal astrocyte networks in function of the local orientation of their processes highlighted the differences in the distribution of retinal astrocyte processes. However, no significant differences between control and hypoxic retinas were found in the area analysed. While these results indicate there was no significant difference between these populations (p >95%), there does appear to be a difference in the degree of organisation (a difference of approximately 93%, p = 0.069).

### Astrocyte morphology in the superior colliculus

**Fig 5:**
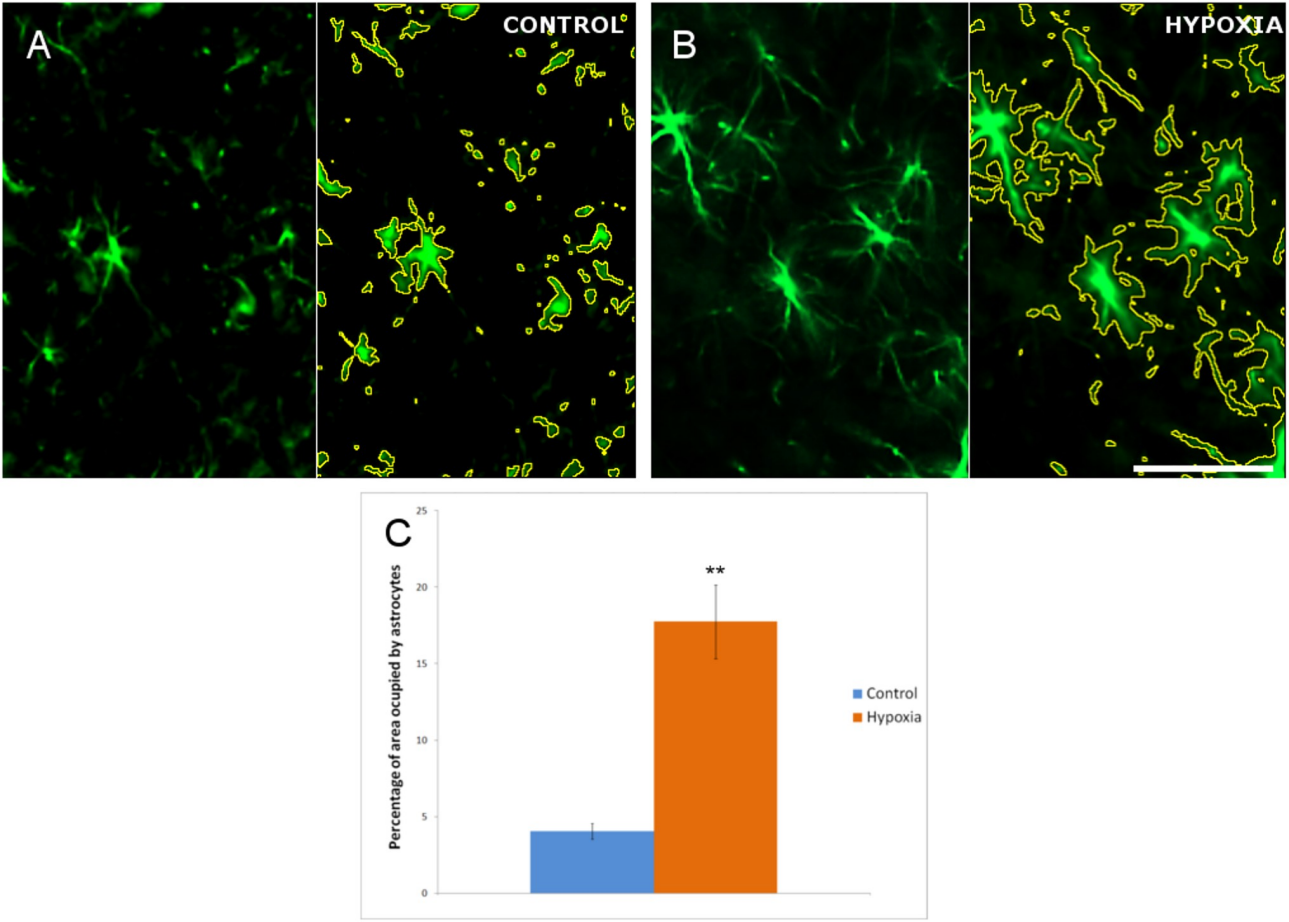
Analysis of astrocytes in the superior colliculus. Images of the control (A) and hypoxic (B) superior colliculus in which the cytoskeleton of the astrocytes is labelled with antibodies against GFAP (green). In A and B the pictures are split by a vertical line where in the right part each astrocyte is defined by a yellow line that represents the area of the cytoskeleton measured by the ImageJ program to quantify the cytoskeleton of the astrocytes. (C) Histogram showing the proportion of the cell occupied by GFAP, the standard errors of the mean are represented as an error bar. Significant differences were found between the control and hypoxic superior colliculus: **p<0.01. Scale bar = 50 μm.

Activation and hypertrophy of astrocytes was evident in the hypoxic superior colliculus (Fig 5), implying the emergence of gliosis. Thus, we cannot rule out that hypoxia induces changes in the number of astrocytes in the superior colliculus. Moreover, the disposition of astrocytes in the superior colliculus meant that we could not use the same method as that employed in the retina to measure the changes in the patterning of their processes. When the area occupied by the astrocyte cytoskeleton was quantified, the mean proportion of the area occupied by the astrocyte’s cytoskeleton in the control superior colliculus was 4.07% (± 0.50), while in the hypoxic superior colliculus it was 17.75% (± 2.41). Moreover, hypoxia induced an increase in astrocyte density of 13.78%, representing a 4.36-fold increment (p = 0.0037).

## Discussion

Experimental studies in suitable animal models commonly provide insights into human neonatal situations. In the present study we have used the pig as an animal model because beside other primates, the porcine retina is the most similar to the human retina among the large mammals [42–44]. Neonatal pigs have been used previously to study brain alterations and to test different drugs [4, 45, 46], and here we have used hypoxic conditions followed by a short recovery to detect early damage, and to compare the effects of hypoxia on the retina and brain.

We found that hypoxia produces significant neuronal apoptosis in the superior colliculus but not in the retina. Moreover, we found significant gliosis of the astrocytes in the superior colliculus but not in the retina. However, in the retina some changes in RGC density in the peripheral retina were found, although they were not significant. The changes described probably reflect the earliest events that result from the hypoxic insult and we cannot rule out that longer recovery times will produce more significant damage in the retina.

Limiting oxygen in the retina contributes to RGC neurodegeneration, which results in a loss of vision and ultimately, blindness. Cell viability can be dramatically compromised by hypoxic stress [24, 47] and we found that the dorsal-periphery of the retina was the area where the RGCs are more vulnerable to hypoxia, even though significant differences were not found. This is consistent with the early events that take place in glaucoma, a neurodegenerative disease caused by elevated intraocular pressure (IOP) that possible causes mild hypoxia [48, 49] and where peripheral RGCs are more sensitive to damage, in accordance with others studies in pigs [50–52], rats [50, 53] and mice [54].

Although several mechanisms of cell death are activated by oxygen depletion in neural tissues [24, 55], changes in apoptotic RGCs were not found and the basal number of active caspase-3 positive cells in the ganglion cell layer was similar in control and hypoxic retinas. By contrast, the proportion of active caspase-3 positive cells after hypoxia increases in the superior colliculus as a whole, and this increases further in the superficial layers of the superior colliculus with respect to the control. Cell death has been described as a natural event in the superior colliculus during development and postnatally [56, 57]. Moreover, hypoxic insult in the developing brain triggers the same apoptotic pathway as that activated during development [58]. The induction of apoptosis following hypoxia is evident just a few hours after insult [59] in brain areas like the cortex and hippocampus of neonatal pigs [34, 45]. Furthermore, the intensity of cell death in the superficial layers of the superior colliculus could reflect the elevated cell density in these layers [60] relative to the rest of the superior colliculus. Thus, in hypoxic conditions, the cells will have more limited access to oxygen in these superficial layers, which could induce them to more readily undergo apoptosis.

Hypoxia induces astrocyte activation in many neurological disorders [61–63]. Reactive astrocytes divide and become hypertrophic, producing long, thick processes [64], as well as overexpressing GFAP [65, 66]. In the superior colliculus, signs of astrocyte activation and reactive gliosis were noted, such as an increase in the surface area occupied by GFAP, although no changes in the orientation of the astrocyte’s prolongations were found in the retinas.

The distinct susceptibility to hypoxia between the retina and the superior colliculus could be explained by the heterogeneity in metabolic and molecular regulation between different areas of the CNS [67] that produce a distinct vulnerability to oxygen depletion, as well as a different degree of neuroprotection [33, 68]. The more severe vulnerability of brain neurons to limited oxygen may be due to the brain tissue failing to up-regulate glycolysis sufficiently in order to compensate for the loss of respiratory ATP. By contrast, the retina has the capacity to metabolize the glucose converted to lactate from glycolysis more efficiently thanks to the Müller glia, only present in the retina [69]. In addition, the stronger resistance to hypoxia in the retina could be due also to the presence of Müller glia cells, which are not present in the brain. Although they share many characteristics with astrocytes, Müller glia have specific functions related to the homeostatic and metabolic support of retinal neurons [32, 70]. Müller cells resist hypoxia and low glucose conditions by activating anaerobic glycolysis and by oxidizing alternative substrates in order to obtain energy in the form of ATP, as well as providing neurons with lactate [69]. Furthermore, surfactant protein A (SP-A) is found in Müller cells, RGCs and astrocytes in the retina, and it is up-regulated during hypoxia. Since SP-A influences neovascularisation, its expression may represent a protective response against systemic inflammation and it may participate in the maintenance of the blood retinal homeostatic barrier [71]. Finally, in transient ischemia we described an increase in brain derived neurotrophic factor (BDNF) in RGCs after ischaemic insult, as well as changes in the neurotrophic p75 receptor in Müller cells [32, 72]. These changes may reflect the neuroprotection in the retina that makes it more resistant to hypoxia in neonates.

The lack of morphological changes in the retina after hypoxia only reflect early events, suggesting that the brain is more vulnerable to hypoxia at these early time points. However, after longer reperfusion times, retinal cells might also be sensitive to such insult. Moreover, given the limited sample size in this study some changes could remain unnoticed, with only major differences becoming evident, such as those in the superior colliculus that appears to be more sensitive to hypoxia. Consequently, a therapeutic window appears to exists in which a neuroprotective action protects the retinal neurons even though the brain has suffered damage.

The results of the present study highlight the differences in the reaction of neurons and astrocytes in different parts of the CNS. As heterogeneous responses to hypoxia were detected in different brain regions, interesting opportunities may be open to design therapeutic and preventative treatments specific to certain areas and structures in the nervous system.

## Supporting information

S1 Fig Scheme of areas of RGC density. Schematic drawing of a pig retina where the four different areas of the retina analysed are represented as black squares: DP, dorsal periphery; DC, dorsal centre; VC, ventral centre; VC, central periphery.
(TIF)

S1 File Supporting information file. File that contains the individual-level data points behind means, and variance measures presented in the results, tables, and figures.
(XLSX)
