# Supplementary figures and images for: Effect of hypoxia on the retina and superior colliculus of neonatal pigs
Source: PLoS One. 2017 Apr 13;12(4):e0175301. doi: 10.1371/journal.pone.0175301 (PMC5391064; doi:10.1371/journal.pone.0175301)

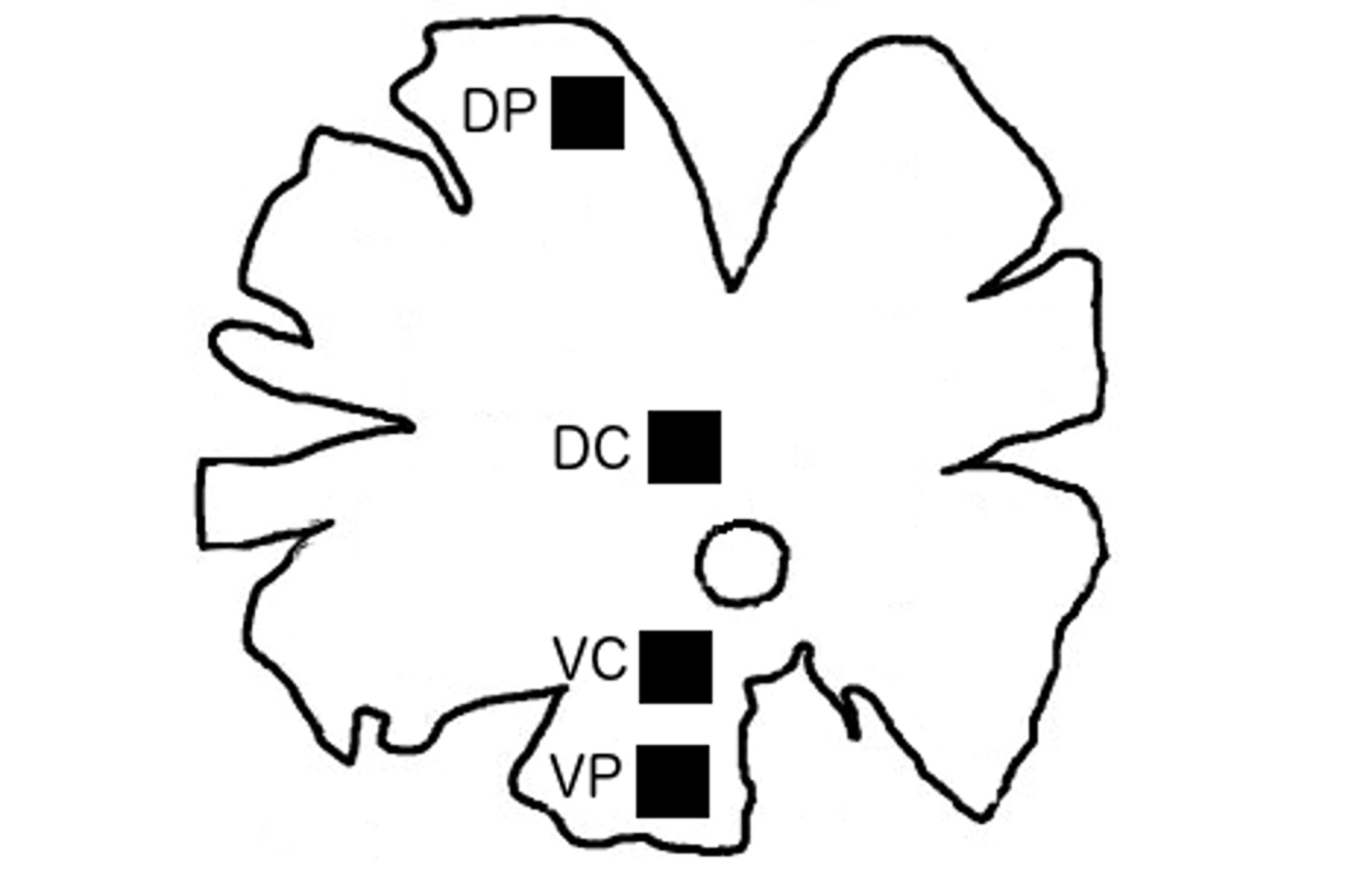

Supplement: S1 Fig — Schematic drawing of a pig retina where the four different areas of the retina analysed are represented as black squares: DP, dorsal periphery; DC, dorsal centre; VC, ventral centre; VC, central periphery. (TIF) [file pone.0175301.s001.tif]
